# Solution-processed transparent blue organic light-emitting diodes with graphene as the top cathode

**DOI:** 10.1038/srep09693

**Published:** 2015-04-20

**Authors:** Jung-Hung Chang, Wei-Hsiang Lin, Po-Chuan Wang, Jieh-I Taur, Ting-An Ku, Wei-Ting Chen, Shiang-Jiuan Yan, Chih-I Wu

**Affiliations:** 1 Graduate Institute of Photonics and Optoelectronics, National Taiwan University, Taipei, Taiwan 106, R.O.C; 2 Graduate Institute of Photonics and Optoelectronics & Department of Electrical and Engineering, National Taiwan University, Taipei 106, Taiwan 106, R.O.C

## Abstract

Graphene thin films have great potential to function as transparent electrodes in organic electronic devices, due to their excellent conductivity and high transparency. Recently, organic light-emitting diodes (OLEDs)have been successfully demonstrated to possess high luminous efficiencies with p-doped graphene anodes. However, reliable methods to fabricate n-doped graphene cathodes have been lacking, which would limit the application of graphene in flexible electronics. In this paper, we demonstrate fully solution-processed OLEDs with n-type doped multilayer graphene as the top electrode. The work function and sheet resistance of graphene are modified by an aqueous process which can also transfer graphene on organic devices as the top electrodes. With n-doped graphene layers used as the top cathode, all-solution processed transparent OLEDs can be fabricated without any vacuum process.

Since the demonstration of the first multi-layer organic light-emitting diodes (OLEDs) in 1987 by Kodak's Tang and Vanslyke^1^, they have attracted tremendous interest due to potential advantages in many applications - for example, displays and lighting. In 1990, Friend *et al.* first introduced the use of a conjugated polymer poly(phenylene-vinylene) (PPV) to produce solution-processed OLEDs^2^. Since then, an explosion of progress has occurred in new processes for manufacture and synthesis of new organic electroluminescent materials. As a result, impressive scientific and technological advances have been achieved, and numerous products have been commercialized.

To date, most OLEDs with small-molecule materials have been fabricated by vacuum deposition, which is easier for building multiple-layer devices and generally more efficient. By contrast, polymer OLEDs are usually built by solution processes, such as spin-coating^3^,^4^, ink-jet printing^5^, screen printing^6^, blade coating^7^, or dip-coating^8^, which enable much cost-effective device manufacture are more suitable for flexible electronic applications^9^. However, it is a challenge to prepare solution-processed OLEDs in multilayer structures due to interface mixing of the different organic layers. In the past decade, although tremendous progress has been made in solution-processed OLEDs, the top electrode has still been mostly fabricated by vacuum deposition. As a consequence, developing solution-processed electrode materials is critical for realizing fully solution-processed OLEDs. Recently, fully printable light-emitting devices have been successfully demonstrated by printing conducting Ag-paste cathodes on top of organic layers^10^. On the other hand, polymer light-emitting electrochemical cells (PLECs) with single-walled carbon nanotube (SWNT) as both anode and cathode have been realized as all-solution-processed light-emitting devices without vacuum deposition^11^. Since low or high work function electrodes are not required for PLECs^12^, many solution-processed conducting materials, such as Ag paste^13^, silver nanowires (AgNWs)^14^, and graphene films^15^, have been applied to achieve fully solution-processed light-emitting devices. Compared with polymer OLEDs, PLECs have relatively low turn-on voltages and elastic selectivity of electrodes. However, some serious disadvantages of PLECs need to be overcome, such as slow response time and short operating lifetimes^16^,^17^.

In this paper, we demonstrate a method of fabricating an all-solution-processed transparent OLED with graphene film as the top cathode without any vacuum deposition process. In previous reports^18^, we have revealed a polymer-free graphene transfer process that enables direct CVD-grown graphene to be transferred from copper to any substrate. Here, this transfer process is employed to enable layer-by-layer transfers of multiple stacked graphene layers with n-type doping. These n-type doped graphene films are incorporated on solution-processed organic layers as a transparent cathode, and fully solution-processed blue-light transparent polymer OLEDs are thus achieved.

## Results

**Figure 1:**
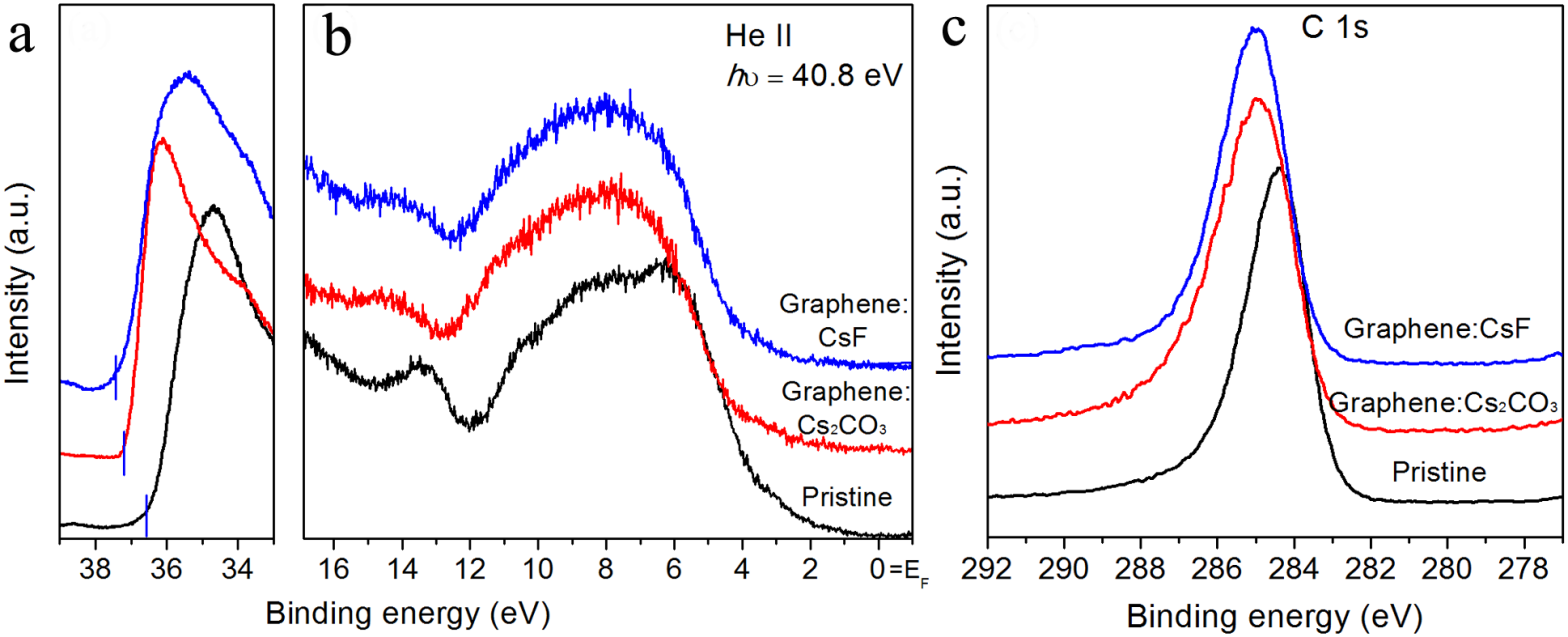
UPS and XPS spectrum of pristine or n-type doped monolayer graphene on SiO_2_. (a) The cut off and (b) valance band spectra of pristine, Cs_2_CO_3_-doped, and CsF-doped monolayer graphene. (c) The XPS spectra of C 1s peak of pristine, Cs_2_CO_3_-doped, and CsF-doped monolayer graphene.

The work function of pristine graphene film is around 4.4 eV^19^, which is too high to reduce the energy barrier of electron injection from the cathode to the organic layers in devices. In order to be used as an effective cathode, the graphene layer is n-type doped during the transfer process to modify the work function. In the last step of transfer, a few drops of CsF or Cs2CO3 solvent are added into the mixture of DI water and IPA solvent before the landing of the graphene film to form n-type doped graphene on the devices, and the concentration of n-type dopant is about 3.3 × 10^−3^ M. Photoemission spectrum experiments are carried out to examine the n-type doping effect on the graphene films. Fig. 1a and b show the results of ultraviolet photoemission spectroscopy (UPS) measurement of pristine monolayer graphene and monolayer graphene doped with CsF or Cs2CO3 on an SiO2 substrate. As shown in Fig. 1a, the cut-off binding energy of pristine graphene is 36.6 eV, and the work function is 4.2 eV, which is too high for a good candidate of a cathode in organic devices. With CsF or Cs2CO3 doping, the cut-off binding energy shifts about 1 eV to higher binding energy, and the same trend can be found in the valance band near the Fermi level (E_F_). As listed in Table 1, the work function can be reduced to 3.2 eV or 3.3 eV with CsF or Cs2CO3 doping using this transfer/doping process.

Moreover, to examine the stability of the n-type doping effect, the graphene samples were kept in the glove box for 2 months. As shown in Table 1, the work functions of both pristine graphene and n-type doped graphene are almost the same as the initial conditions. This reveals that the n-type doping effect is very stable with this method.

On the other hand, Fig. 1c shows the X-ray photoemission spectroscopy (XPS) spectra of the C 1s core level of pristine as well as CsF- or Cs2CO3-doped graphene. As revealed by the XPS results, the C 1s peak is shifted to higher binding energy from 284.3 eV to 285.1 eV and 285.0 eV by doping with CsF and Cs2CO3, respectively. This supports the assumption that the electron transfer between the graphene and CsF or Cs2CO3 leads to n-type doping of graphene films. On the other hand, the Cs core-level signal is also observed in the n-type doped graphene samples with atomic concentrations of Cs of 4.5% and 4% for CsF- and Cs2CO3-doped graphene, respectively.

**Figure 2:**
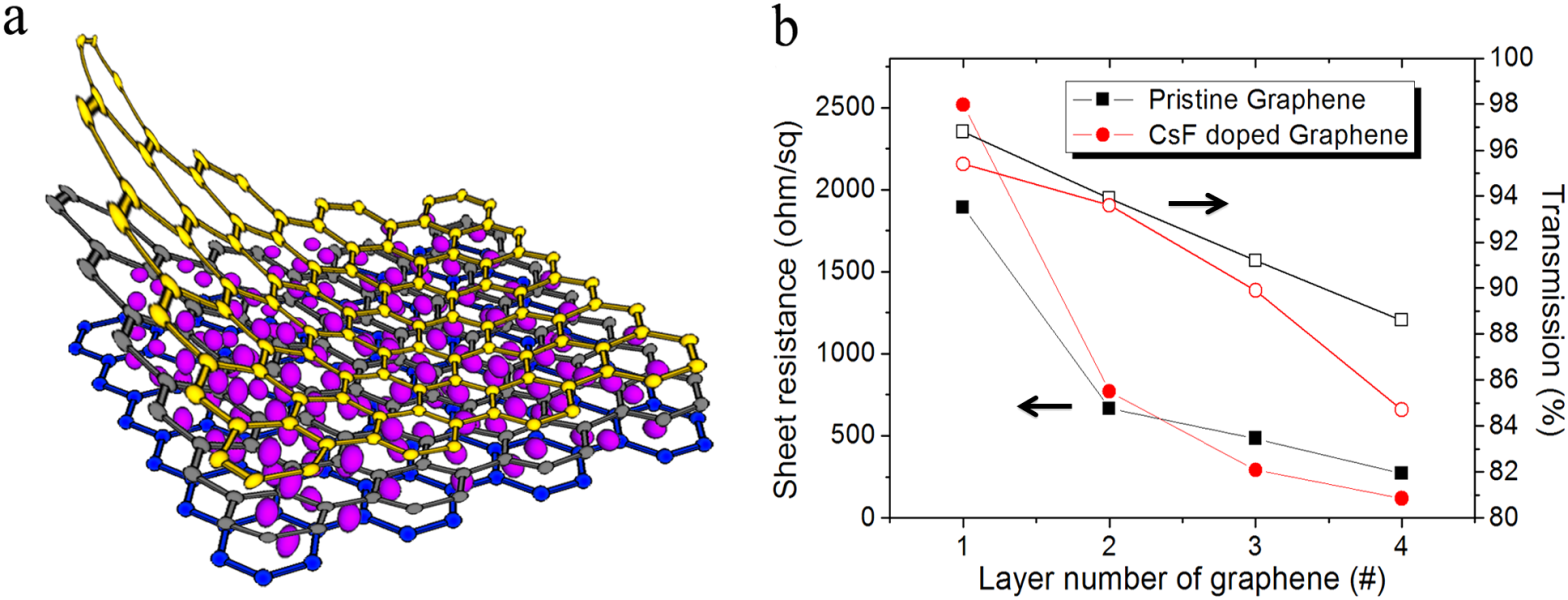
The schematic illustration and characteristics of n-type doped graphene films. (a) The schematic illustration of CsF-doped graphene multilayer. (b) Sheet resistance and transmittance at 500 nm of pristine (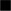) and CsF-doped (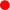) graphene with number of layers.

To enhance the electrical properties of graphene as applied in organic devices, we increase the number of graphene layers. Moreover, to maintain the lower work function properties, graphene is n-doped during every transfer process. As shown in Fig. 2a, Cs atoms are intercalated between every graphene layer to form n-type doped multilayer graphene films. The sheet resistance and transmittance with varying numbers of graphene layers on glass are investigated as shown in Fig. 2b. The transmittances at a wavelength of 500 nm of one-, two-, three- and four-layer pristine graphene samples are 96.8%, 93.9%, 91.2%, and 88.6%, respectively. At the same time, the sheet resistance values of one- to four-layer pristine graphene films are 1892, 666, 484, and 270 ω/sq, respectively. As the number of layers increases, the transmittance and sheet resistance decrease. The optical transmittance of graphene films is reduced by around 2.2–2.3% for each additional layer, and the overall conductivity of the graphene films increases as the number of stacked layer increase^20^,^21^. On the other hand, the transmittances of one- to four-layer CsF-doped graphene films are 95.4, 93.6, 89.9, and 84.9%, respectively. It is noted that the transmittance drops to 84.9% with a four-layer CsF-doped graphene film, a value similar to that reported for multilayer graphene doped with 2,3,5,6-Terafluoro-7,7,8,8-teracyanoquinodimethane (F4-TCNQ)^22^. The sheet resistances of one- to four-layer CsF-doped graphene films are 2515, 770, 290, and 118 ω/sq, respectively. The sheet resistance of CsF-doped monolayer graphene is higher than that of pristine monolayer graphene because a multilayer graphene structure is needed to form layer-by-layer intercalations of graphene and Cs atoms for effective doping. Therefore, conductivity is distinctly enhanced with increasing numbers of graphene layers.

**Figure 3:**
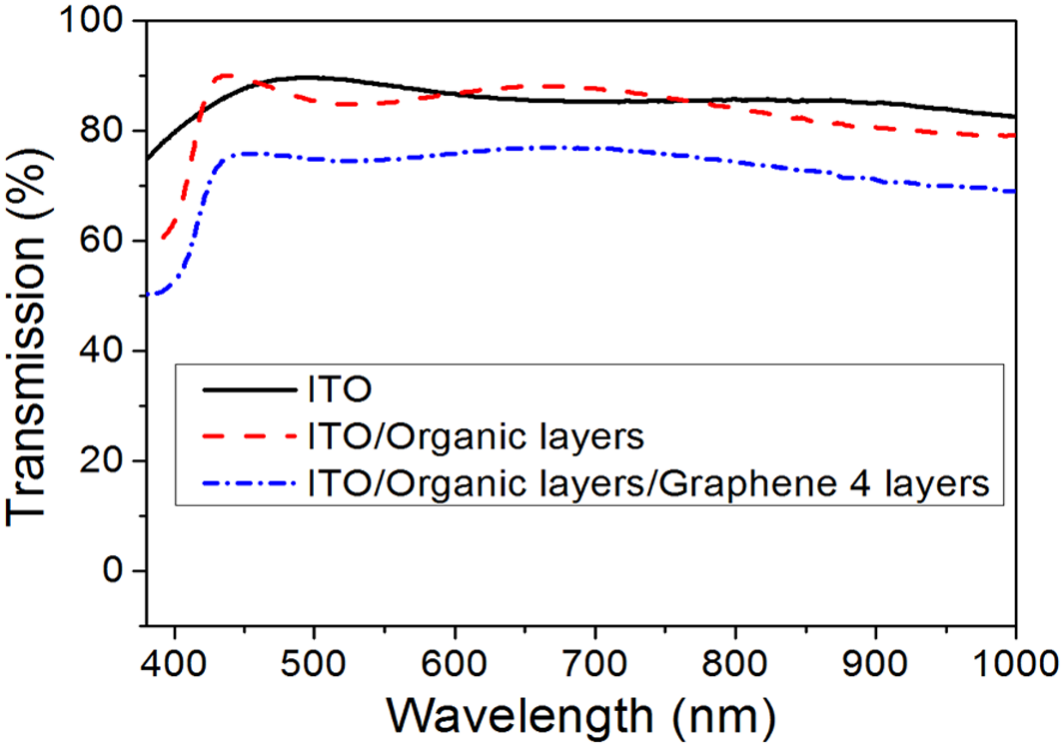
The transmittance of glass/ITO, glass/ITO/four organic layers, and glass/ITO/four organic layers/4-layer graphene:CsF.

After demonstration of the capacity of n-type doped graphene as a cathode, the CsF-doped multilayer graphene films are used as the top cathode in solution-processed multilayer OLEDs. To begin with, the transmittance of the device is investigated. As shown in Fig. 3, the transmittance of ITO/glass is about 90% in the region of visible light. After the deposition of organic layers, including 1,4,5,8,9,11-hexaazatriphenylene hexacarbonitrile (HAT-CN), thermally polymerized 2,7-disubstituted fluorene-based triaryldiamine (VB-FNPD), an emitting layer, and poly[(9,9-bis(30-(N,N-dimethylamino)propyl)-2,7-fluorene)alt-2,7-(9,9-dioctylfluorene)] (PFN), the transmittance is around 90%, which is almost no reduction from the ITO/glass substrate. Although there is an obvious drop of transmittance after the deposition of multilayer graphene on top of the organic layers, the transmittance remains around 75% in the region of visible light.

**Figure 4:**
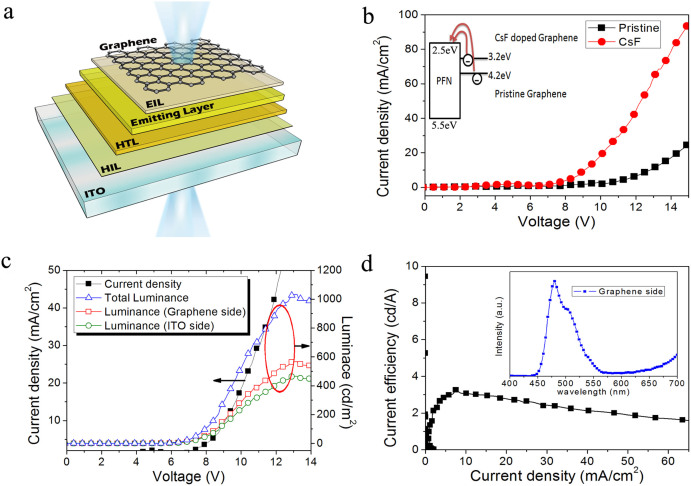
Device structure and performance of all-solution blue-light transparent polymer OLEDs with CsF-doped multilayer graphene as top cathode. (a) The device structure: ITO/HAT-CN/VB-FNPD/PVK:Firpic/PFN:CsF/multilayer graphene, (b) The current density versus voltage (J-V) curves of devices with (▪) 4-layer pristine graphene and (•) 4-layer CsF-doped graphene as cathode. The inset shows the schematic illustration of electron injection from pristine graphene or CsF-doped graphene. (c) The current density-voltage-luminance (J-V-L) curve of the transparent polymer OLEDs, (d) Luminance efficiency verse current density characteristics. The inset shows electroluminescence spectra of the blue-light transparent polymer OLEDs from graphene cathode side.

Fig. 4a shows the device structure of fully solution-processed transparent organic blue-light-emitting devices with CsF-doped graphene layers as the top cathode: ITO/HAT-CN/VB-FNPD/poly(N-vinylcarbazole)(PVK):bis(4′,6′-difluorophenylpyri-dinato) iridium(III) picolinate(Firpic) (10 wt%)/PFN:CsF/Graphene:CsF. As described in the Methods section,graphene films are transferred onto the top of organic layers with a polymer-free method. These organic films need to be immersed in DI water during the graphene transfer process. Consequently, HAT-CN is chosen here as the HIL to replace commonly-used PEDOT:PSS, which would dissolve in DI water. It has been reported that HAT-CN has a more stable interface^23^,^24^ and only dissolves in acetone^25^; therefore, HAT-CN is the better HIL here to withstand immersion in DI water. In addition, the cross-linkable derivative of NPD, VB-FNPD, is used here as HTL which displays remarkable ambient stability^26^,^27^. Moreover, the alignment of electronic energy levels at HAT-CN/VB-FNPD reveals that the interface between HAT-CN and VB-FNPD layers can be assumed as ohmic contact, which improves hole injection^25^. Before the deposition of multilayer graphene as cathode, the PFN is used as an EIL to prevent exciton quenching at the interface between the emitting layer and graphene cathode^28^,^29^. The mixture of methanol solution and water is used for PFN to increase the brightness^30^.

To reveal the n-type doping effect of graphene in electron injection from the cathode, pristine multilayer graphene and CsF-doped multilayer graphene are applied to OLED devices as top cathodes. The current density to voltage (J-V) of the devices is shown in Fig. 4b. Compared with pristine graphene as cathode, the current density with CsF-doped graphene as cathode is noticeably enhanced. This enhancement is directly related to the lower work function of graphene with n-type doping, and it can be explained by the inset in Fig. 4b. The injection barrier of electrons from the cathode is reduced with CsF-doped graphene (3.2 eV), and more electrons can be injected from graphene into the organic layer to increase current density. This enhancement is consistent with the previous UPS results.

The current density-voltage-luminance (J-V-L) characteristics and efficiencies of the all-solution blue-light OLEDs fabricated with CsF-doped multilayer graphene as the top cathode are shown in Fig. 4c and 4d. The maximum brightness of this transparent OLED is 1034 cd/m^2^ at 13 V, and the maximum current efficiency is 3.1 cd/A at 8.3 mA/cm^2^. Compared with the typical device with thermally deposited Al as the top cathode, OLEDs with graphene as the top cathode show poorer performance. This can be attributed to poorer contact between the organic layer and the graphene film cathode, resulting in fewer electrons from cathode to organic layer.

**Figure 5:**
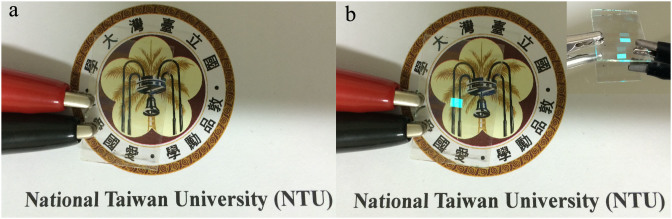
Photographs of all-solution blue-light transparent polymer OLEDs. The photographs show the device (a) before and (b) during operation with active size: 2 × 3 mm^2^. The inset shows the photograph of a working blue-light polymer OLED in front of a mirror, showing emission from both surfaces.

The electroluminescence (EL) spectra of the blue-light transparent OLEDs on the graphene cathode side are shown in the inset of Fig. 4d, and the maximum emission wavelength of the blue-light OLEDs is 477 nm, which is consistent with reported spectra^31^. Fig. 5a and 5b present photographs of the transparent blue-light device before and during operation. The inset in Fig. 5b shows a photograph of a working blue-light transparent OLED in front of a mirror, which shows emission from both surfaces. Although the efficiency and brightness of this transparent device may be lower than other reported devices, we demonstrate an approach to realize fully solution-processed transparent OLEDs without vacuum deposition.

To be a good candidate as a cathode in an organic device, graphene should be n-type doped to reduce the electron injection barriers from cathode to organic layers. A new polymer-free transfer method has been demonstrated to be an efficient way to modify the work function and sheet resistance of graphene, and n-type doped multilayer graphene films obtained using this method can be used as cathodes in OLEDs. Moreover, we have provided a way to produce a transparent OLED with truly all-solution processes, including multilayer graphene as the top electrode. A fully solution-processed transparent blue-light multilayered OLED with n-doped graphene layers used as the top cathode has been produced without any vacuum process. Although the efficiency and brightness of this transparent device may be lower due to the poorer contact between the organic layer and the graphene film cathode. With this transfer method, graphene electrodes can be used in a wide variety of organic optoelectronics with more efficient doping and simple transfer techniques.

## Methods

### Graphene transfer and doping

Single-layer graphene films were synthesized on copper foils by thermal CVD. To form multilayer graphene films, each graphene monolayer was transferred, with or without n-type doping, onto other graphene films. The process of preparation of multilayer graphene films was as follows: A clean Petri dish was filled with 0.1 M ammonium persulphate solution ((NH_4_)_2_S_2_O_8_) as etchant, and a thin graphite holder with a diameter of 2 cm was then carefully placed at the etchant air boundary, serving as a confinement area for the graphene monolayer on a copper substrate and preventing it from attaching to the edge of the holder. After the copper was etched, the monolayer graphene film was floating on the surface of the solution and the etchant was replaced by a mixture of DI water and IPA solution. After the etchant was totally replaced by the water and IPA, the substrate was placed just below the floating graphene in the solution. The solution was then pulled out with a syringe to lower the graphene onto the substrate. For the n-type doping graphene, a few drops of n-type dopant (CsF or Cs2CO3) were added to the mixture before the extraction of solution. After landing on the substrate, the sample was then heated at 60°C in nitrogen for 10 min to dry the graphene sheets. More details of the experimental setup for the preparation of multilayer graphene films which can be applied to any substrate can be found in our previous report.

### Photoemission spectroscopy (PES) measurements

Pristine or doped monolayer graphene film was transferred onto SiO_2_ substrates for photoemission experiments, and monolayer/multilayer graphene films were transferred onto SiO_2_ or glass for sheet resistance or transmission measurements. Photoemission experiments were carried out by ultraviolet photoemission spectroscopy (UPS) and X-ray photoemission spectroscopy (XPS) in an ultra-high vacuum (UHV) chamber with a base pressure of 10^−10^ torr. The photon energies of UPS were 21.2 eV and 40.8 eV for He I and He II respectively, and the resolution was around 0.15 eV.

### Organic film growth and device fabrication

The solution-processed blue Ph-OLEDs were fabricated based on a phosphorescent emission layer (EML) doped with a blue triplet emitter. These Ph-OLEDs were fabricated on ITO-coated glass substrates with a sheet resistance of 15ω/square. The ITO substrate was UV-ozone treated before fabrication of the device, and the structure of the device was ITO/HAT-CN/VB-FNPD/PVK:Firpic:OXD-7/PFN/n-type doped graphene. A solution of 1,4,5,8,9,11-hexaazatriphenylene hexacarbonitrile (HAT-CN) (3 mg/ml) was prepared with 2-propanone (acetone) as the solvent, and the solution was spin-coated onto the substrates to form about 5 nm as the hole injection layer (HIL). A thermally polymerized 2,7-disubstituted fluorene-based triaryldiamine (VB-FNPD) was dissolved in chlorobenzene (10 mg/ml) and was spin-coated onto the HAT-CN surface to form a cross-linked hole transport layer (HTL). After that, the blue-light emission layers (EMLs), which consisted of a mixture of poly(N-vinylcarbazole) (PVK), and bis(4′,6′-difluorophenylpyri-dinato) iridium(III) picolinate (FIrpic), were spin-coated onto the VB-FNPD layer from the chlorobenzene solution with a ratio of 9:1, and annealed at 90°C for 30 min to remove the residual solvent. The alcohol/water-soluble polymer, poly[(9,9-bis(30-(N,N-dimethylamino)propyl)-2,7- fluorene)alt-2,7-(9,9-dioctylfluorene)] (PFN) was incorporated as an electron-injection layer on top of the emitting layer by spin-coating. The PFN solution was first prepared by dissolving PFN in methanol (4 mg/ml). Second, the PFN solution was mixed with water with a volume ratio of 3:1. The mixed solvent was blended with a 0.1% CsF solution at a volume ratio of 8:1, and finally, the blended solution was spin-coated onto the emitting layer to form a 30 nm-thick film. After deposition of the PFN layer, the n-type doping multilayer graphene was transferred onto the PFN layer as the top cathode. After the landing of the multilayer graphene, the OLED was annealed at 80°C for 20 min. All the organic layers were deposited in a glove box except the PFN layer.

### Device characterization

After fabrication of the device, the current density-voltage-luminance (*J-V-L*) measurement was carried out immediately in the atmosphere. The *J-V* and *L-V* characteristics were simultaneously analyzed by a Keithley 2400 and by photodiodes. The electroluminescence (EL) spectrum was recorded with PR-650 Spectra Colorimeter. The transmittances were measured by V-670 UV-Vis-NIR Spectrophotometer (Jasco *Inc*.).All measurements were performed at room temperature in ambient air.

## Author Contributions

J.H.C. designed and performed the experiments with data analysis; W.H.L. and J.I.T. helped with the analysis of graphene films. P.C.W., T.A.K. and W.T.C. helped with the device fabrication. S.J.Y. laid out the figures in the text. C.I.W. provided the advice on and coordinated the experiments. J.H.C. and C.I.W. wrote the manuscript.

## Figures and Tables

**Table 1:** The work function initially and after 2 months stored in glove box, of pristine, Cs_2_CO_3_-doped, and CsF-doped monolayer graphene

	Pristine	G:CsF	G:Cs_2_CO_3_
Initial	4.2 eV	3.2 eV	3.3 eV
2 months	4.2 eV	3.3 eV	3.4 eV

## References

[b1] Tang C. W. & VanSlyke S. A. Organic electroluminescent diodes. Appl. Phys. Lett. 51, 913–915 (1987).

[b2] Burroughes J. H. *et al.* Light-emitting diodes based on conjugated polymers. Nature 347, 539–541 (1990).

[b3] Friend R. H. *et al.* Electroluminescence in conjugated polymers. Nature 397, 121–128 (1999).

[b4] Müller C. D. *et al.* Multi-colour organic light-emitting displays by solution processing. Nature 421, 829–833 (2003). 12594509 10.1038/nature01390

[b5] Hebner T. R., Wu C. C., Marcy D., Lu M. H. & Sturm J. C. Ink-jet printing of doped polymers for organic light emitting devices. Appl. Phys. Lett. 72, 519–521 (1998).

[b6] Pardo D. A., Jabbour G. E. &Peyghambarian N. Application of screen printing in the fabrication of organic light-emitting devices. Adv. Mater. 12, 1249–1252 (2000).

[b7] Ko L. C. *et al.* Multi-layer organic light-emitting diodes processed from solution using phosphorescent dendrimers in a polymer host. Org. electronics 11, 1005–1009 (2010).

[b8] Yimsiri P. &Mackley M. R. Spin and dip coating of light-emitting polymer solutions: matching experiment with modelling. Chem. Eng. Sci. 61, 3496–3505 (2006).

[b9] Gustafsson G. *et al.* Flexible light-emitting diodes made from soluble conducting polymer. Nature 11, 477–479 (1992).

[b10] Zeng W. *et al.* Polymer light-emitting diodes with cathodes printed from conducting Ag paste. Adv. Mater. 19, 810–814 (2007).

[b11] Yu Z. *et al.* Highly flexible polymer light-emitting devices using carbon nanotubes as both anodes and cathodes. SPIE 1, 011003 (2011).

[b12] Pei Q., Yu G., Zhang C., Yang Y. & Heeger A. J. Polymer light-emitting electrochemical cells. Science 269, 1087–1088 (1995). 10.1126/science.269.5227.1086 17755530

[b13] Liang J., Li L., Niu X., Yu Z. & Pei Q. Fully solution-based fabrication of flexible light-emitting device at ambient conditions. J. Phys. Chem. C 117, 16632–16639 (2013).

[b14] Liang J., Li L., Niu X., Yu Z. & Pei Q. Elastomeric polymer light-emitting devices and displays. Nat. Photonics 7, 818–824 (2013).

[b15] Matyba P. *et al.* Graphene and mobile ions: the key to all-plastic, solution-processed light-emitting devices. ACS Nano 4, 637–642 (2010). 20131906 10.1021/nn9018569

[b16] Yu G., Cao Y., Andersson M., Gao J. & Heeger A. Polymer light-emitting electrochemical cells with frozen p-i-n junction at room temperature. J. Adv. Mater. 10, 385–388 (1998).

[b17] Shin J. H., Xiao S., Fransson Å. & Edman L. Polymer light-emitting electrochemical cells: frozen-junction operation of an “ionic liquid” device. Appl. Phys. Lett. 87, 043506 (2005).

[b18] Lin W. H. *et al.* A direct and polymer-free method for transferring graphene grown by chemical vapor deposition to any substrate. ACS Nano. 8, 1784–1791 (2014). 24471977 10.1021/nn406170d

[b19] Han T. H. *et al.* Extremely efficient flexible organic light-emitting diodes with modified graphene anode. Nat. Photonics 6, 105–110 (2012).

[b20] Li X. *et al.* Transfer of large-area graphene films for high-performance transparent conductive electrodes. Nano Lett. 9, 4359–4363 (2009). 19845330 10.1021/nl902623y

[b21] Bae S. *et al.* Roll-to-roll production of 30-inch graphene films for transparent electrodes. Nat. Nanotechnol. 5, 574–578 (2010). 20562870 10.1038/nnano.2010.132

[b22] Song J. *et al.* A general method for transferring graphene onto soft surfaces. Nat. Nanotechnol. 8, 356–362 (2013). 23624698 10.1038/nnano.2013.63

[b23] Chang J. H. *et al.* Stability improvement of organic light emitting diodes by the insertion of hole injection materials on the indium tin oxide substrate. J. Appl. Phys. 115, 124510 (2014).

[b24] Kim H. J. *et al.* Molecular alignment and nanostructure of 1,4,5,8,9,11- hexaazatriphenylene-hexanitrile (HATCN) thin films on organic surfaces. J. Mater. Chem. C 1, 1260–1264 (2013).

[b25] Lin H. W., Lin W. C., Chang J. H. & Wu C. I. Solution-processed hexaazatriphenylene hexacarbonitrile as a universal hole-injection layer for organic light-emitting diodes. Org. Electron. 14, 1204–1210 (2013).

[b26] Lin C. Y. *et al.* A thermally cured 9,9-diarylfluorene-based triaryldiamine polymer displaying high hole mobility and remarkable ambient stability. J. Mater. Chem. 19, 3618–3623 (2009).

[b27] Du B. S. *et al.* Os(II) based green to red phosphors: A great prospect for solution-processed, highly efficient organic light-emitting diodes. Adv. Funct. Mater. 22, 3491–3499 (2012).

[b28] Wu H. *et al.* Efficient electron injection from bilayer cathode consisting of aluminum and alcohol-/water-soluble conjugated polymers. Adv. Mater. 16, 1826–1830 (2004).

[b29] Jiamg Z. *et al.* Highly efficient, solution processed electrofluorescent small molecule white organic light-emitting diodes with a hybrid electron injection layer. ACS Appl. Mater. Interface 6, 8345–8352 (2014). 10.1021/am501207g 24840940

[b30] Zhang Y., Huang F., Chi Y. & Jen A. K. Y. Highly efficient white polymer light-emitting diodes based on nanometer-scale control of the electron injection layer morphology through solvent processing. Adv. Mater. 20, 1565–1570 (2008).

[b31] Yook K. S. & Lee J. Y. Solution processed high efficiency blue and white phosphorescent organic light-emitting diodes using a high triplet energy exciton blocking layer. Org. Electron. 12, 1293–1297 (2011).

